# Fetal growth is associated with CpG methylation in the P2 promoter of the *IGF1* gene

**DOI:** 10.1186/s13148-018-0489-9

**Published:** 2018-04-19

**Authors:** Catherine Le Stunff, Anne-Laure Castell, Nicolas Todd, Clémence Mille, Marie-Pierre Belot, Nathalie Frament, Sylvie Brailly-Tabard, Alexandra Benachi, Delphine Fradin, Pierre Bougnères

**Affiliations:** 1 Institut National de la Santé et de la Recherche Médicale U1169, Bicêtre Hospital, Paris Sud University, Le Kremlin-Bicêtre, France; 2 Service de Médecine des Adolescents, Bicêtre Hospital, Paris Sud University, Le Kremlin-Bicêtre, France; 3 Service de BiologieMoléculaire et Hormonologie, Bicêtre Hospital, Paris Sud University, Le Kremlin-Bicêtre, France; 4 Service de Gynécologie-Obstétrique, Antoine Béclère Hospital, Paris Sud University, Clamart, France; 5 CRCINA, INSERM U1232, Université de Nantes, Nantes, France

**Keywords:** Newborn, Growth, Epigenetics, DNA methylation

## Abstract

**Background:**

There are many reasons to think that epigenetics is a key determinant of fetal growth variability across the normal population. Since *IGF1* and *INS* genes are major determinants of intrauterine growth, we examined the methylation of selected CpGs located in the regulatory region of these two genes.

**Methods:**

Cord blood was sampled in 159 newborns born to mothers prospectively followed during their pregnancy. A 142-item questionnaire was filled by mothers at inclusion, during the last trimester of the pregnancy and at the delivery. The methylation of selected CpGs located in the promoters of the *IGF1* and *INS* genes was measured in cord blood mononuclear cells collected at birth using bisulfite-PCR-pyrosequencing.

**Results:**

Methylation at *IGF1* CpG-137 correlated negatively with birth length (*r* = 0.27, *P* = 3.5 × 10^−4^). The same effect size was found after adjustment for maternal age, parity, and smoking: a 10% increase in CpG-137 methylation was associated with a decrease of length by 0.23 SDS.

**Conclusion:**

The current results suggest that the methylation of *IGF1* CpG-137 contributes to the individual variation of fetal growth by regulating *IGF1* expression in fetal tissues.

**Electronic supplementary material:**

The online version of this article (10.1186/s13148-018-0489-9) contains supplementary material, which is available to authorized users.

## Background

Since human neonates are about the same size as the birth canal, size at birth has to be a tightly regulated trait. It is controlled by a number of interacting genetic and environmental factors. Among these factors, IGF1 (insulin-like growth factor-1), IGF2 (insulin-like growth factor-2), and insulin are three key players. The expression of *IGF1* and *IGF2* genes in fetal tissues and placenta is essential for fetal growth, as indicated by studies of knock-out mice and genetic defects in human. Although *IGF2* is more abundantly expressed in fetal serum and tissues than *IGF1*, IGF1 is more closely associated with fetal growth in a majority of species. Insulin secreted by the fetal ß cells is also a major growth factor during intrauterine life [1].

In animal studies, *IGF1* gene ablation reduces fetal weight [2, 3]. Fetal IGF1 promotes tissue growth by stimulating anabolic events and DNA synthesis. Circulating IGF1 is most often decreased in animal models of fetal growth restriction, as well as in human fetal growth restriction. Deletions or loss-of-function mutations in the *IGF1* gene cause intrauterine and postnatal growth retardation [4–7]. In mice, the deletion of the *IGF2* gene reduces fetal growth [2]. The effects on fetal growth are additive to those of IGF1. *IGF2* is an imprinted gene. The expression of *IGF2* maternal allele is silenced during fetal life, leading to fetal growth restraint when the paternal allele is mutated or deleted. Epimutations and molecular alterations of the human chromosomal region 11p15.5, which harbors *IGF2* [8], and paternally inherited nonsense mutations of the *IGF2* gene [9] are associated with the Silver–Russell syndrome, a syndromic growth-retardation disorder. Heterozygous mutations in the *INS* gene are associated with a reduction of fetal growth [10].

While monogenic defects in the *IGF1*, *IGF2*, and *INS* genes demonstrate the major role of these genes for fetal growth, the genetics of the individual variability in birthweight of normal neonates is complex, as for most quantitative traits, and relies on numerous polymorphic variations of the genome. Birth size is a multifactorial phenotype resulting from many processes and gene expression patterns operating during fetal development. Genetic variation is said to account for 38–80% of birth weight variance, with a considerable variability in the estimates [11, 12]. A genome-wide association study found 60 loci to be associated with birth weight at genome-wide significance [13]. Despite their implication in monogenic disorders of fetal growth, neither *IGF1* nor *INS* loci were among these 60 loci, but pathways involved in insulin and IGF signaling were said to be involved. As pointed out for most quantitative traits studied in humans, the genetics of birth size face the missing heritability problem. Indeed, in contrast with the estimates of birth weight heritability [12, 14], the 60 variants most recently identified could only explain 2% of the variance in birth weight [13]. When SNPs at the *INS* [15, 16] and *IGF1* loci [17, 18] were examined specifically as candidates for their contribution to the variation of size at birth, they showed inconsistent results [15, 19].

The genotype of the fetus is not the sole determinant of fetal growth. It is likely that maternal genes that regulate the environment of the womb are important determinants of birth size. Maternal environment also has a major influence, although most of the environmental factors and mechanisms of reduced fetal growth remain largely unknown. Non-inherited maternal factors found to influence birth size include parity, mother weight at birth and before pregnancy, weight gain of pregnancy, and maternal smoking, but cannot explain the vast majority of cases of intrauterine growth retardation (IUGR) [20]. Maternal nutrition, in industrialized populations seems to have only a small effect on placental and birth weights [21]. Indeed, even in affluent societies where mothers are well nourished, many children are still born with unexplained small fetal size. On the other side, extreme maternal nutrition, such as the Dutch Hunger Winter famine, is associated with a reduced birth weight [22].

As for all quantitative traits, epigenetic influences are likely to contribute to the regulation of birth size at the individual level. Few studies have yet explored the epigenetics of fetal growth in humans. To our knowledge, the only epigenome-wide association study carried out yet found that among the 485,577 CpGs analyzed in cord blood by the 450K Infinium BeadChip, 19 CpGs were significantly associated with birthweight in a large population-based cohort [23]. Another study of methylation (performed on cord blood and placenta) restricted to growth-related genes found no association between intrauterine growth retardation and IUGR and the methylation of CpGs at the *IGF1*, *IGF2*, and *INS* loci (note that this study investigated only 3 CpGs located within the *IGF1* P1 promoter) [24].

The current study investigates the relationship between birth length and the methylation of specific CpGs of the *IGF1* and *INS* genes. These CpGs located within promoters were selected because their level of methylation was known to control gene expression. This is the case for *IGF1* CpG-137, whose methylation is associated with *IGF1* gene transcription [25], circulating IGF1 [25] and childhood growth [25, 26]. This is also the case for CpG-180 in the *INS* promoter region, which influences *INS* gene transcription [27] and is associated with SNPs as rs689 [28] known to predict birth size to some degree [15]. We have not explored a potential association of birth length with CpG methylation on the non-imprinted allele of *IGF2* because we did not have access to parental genotypes.

## Methods

### Mothers

One hundred and fifty-nine women of European ancestry aged 20–40 years, with a singleton pregnancy, were recruited at Antoine Béclère Maternity. The clinical characteristics of the 159 newborns are presented in Table 1. All were healthy neonates born after 37 weeks of amenorrhea. None had IUGR, defined as a weight or length below the 10th percentile for its gestational age [29]. Children born before 36 weeks of gestation were not included. Clinical data and biological samples were collected at inclusion (< 3 months of pregnancy) and around delivery. The main characteristics of the mothers are presented in Table 1. All mothers filled a 142-item questionnaire. All mothers were healthy and had a normal nutrition; none consumed alcohol or drugs during their pregnancy. All protocols were agreed by French ethic boards (CODECOH DC-2013-2017, CPP CO-14-001, CCTIRS no. 14-124bis, CNIL no. 914,253). Cord blood samples were taken within minutes of delivery, immediately refrigerated at 4 °C and transported to laboratory within 24 h.

**Table 1:** Main characteristics of the studied newborns and mothers (mean ± sd)

*N*	159
Sex (M/F)	84/75
Term of birth (weeks)	39.8 ± 1.1
Birth length (cm)	49.4 ± 1.4
Birth length (SDS)	− 0.43 ± 0.6
Birth weight (g)	3355 ± 330
Birth weight (SDS)	0.19 ± 0.7
Midparental height (cm)	169 ± 7
IGF1 (ng/ml)	50 ± 19
Insulin (μU/ml)	4.5 ± 4
Mother’s age (years)	32.1 ± 3.9
Mother weigh gain (kg)	10.9 ± 4
Smoking (%)	6
Primaparous (%)	35
Folate supplement before conception (%)	32
Folate supplement during pregnancy (%)	79

### Isolation of genomic DNA and bisulfite genomic conversion

Cord blood mononuclear cells (CBMC) were isolated from fresh blood using a density gradient. Five millimeters of fresh blood was mixed with 5 ml of NaCl 154 mM, and 5 ml of Lymphoprep solution (Eurobio, Paris, France) was added to the diluted blood and centrifuged for 20 min at room temperature at 800 g. After centrifugation, the interphase containing CBMC was carefully aspirated and the cells were mixed with NaCl. The cell suspension was centrifuged at 300 g, and the cell pellet washed with PBS.

Nucleic acids were extracted from CBMC using Gentra Puregene blood kit (Qiagen, Hilden, Germany). Four hundred nanograms of genomic DNA was treated with EZ DNA Methylation-Gold Kit, according to the manufacturer’s protocol (Zymo Research Corporation, CA, USA). A bisulfite-PCR-pyrosequencing technique was used to measure the CG methylation [25]. We improved the resolution of this method from a handful of bases to up to 100 nucleotides, with the ability to quantify methylation in the same sample of blood cells with a coefficient of variation (sd/mean) as little as 1–5% [25, 28].

### Pyrosequencing-based bisulfite PCR analysis

CpGs are denominated according to their position versus each promoter transcription start site (TSS). At the *IGF1* locus, we studied 3 CpGs (-1044, -960, -919) located within the P1 promoter and 5 CpGs (-232, -224, -218, -207, -137) located within the P2 promoter (Additional file 1: Figure S1). We previously have shown that the methylation of the P1 promoter does not influence *IGF1* gene expression [25]. At the INS locus, we studied 2 CpGs (-206 and -180) proximal to the TSS (Additional file 1: Figure S1). The bisulfite-treated genomic DNA was PCR-amplified using unbiased primers (Additional file 2: Table S1) and performed quantitative pyrosequencing using a PyroMark Q96 ID Pyrosequencing instrument (Qiagen). Pyrosequencing assay was designed using MethPrimer (htpp://urogene.org/methprimer/index1.html) [25]. Biotin-labeled single stranded amplicon was isolated according to protocol using the Qiagen Pyromark Q96 Work Station and underwent pyrosequencing with 0.5 μM of sequencing primer. The methylation percent for each CpG within the target sequence was calculated using PyroQ CpG Software (Qiagen).

### Genotyping of SNPs at the IGF1 and INS loci

Genotyping of SNP rs35767 at the *IGF1* gene locus was performed with TaqMan allelic discrimination using Biosystems 7500 Fast Real-Time PCR System (Applied Biosystems, Courtaboeuf, France). SNP genotyping assay (ID: C_7999146_10) was purchased from Life technologies (Saint Aubin, France).

Genotyping of SNP rs689 at the *INS* gene locus was determined by the analysis of PCR products [30]. PCR amplification was in 96-well microliter plates, each 50 μl reaction contained DNA (100 ng), MgCl_2_ (1.5 mM), 10× reaction Buffer (Thermo Scientific), dNTPs (2.0 mM each), primers (1 μM each), and Taq Polymerase (1.25 U, Thermo Scientific, Saint Aubin, France). Amplified PCR products were digested with 1 unit of Hph1 enzyme (Thermo Scientific, Saint Aubin, France).

For genotyping quality, negative controls were included in each PCR plate. Twenty percent of samples were analyzed as duplicate for genotyping determination. The Hardy-Weinberg equilibrium (HWE) was calculated by computing the test for deviations in HWE and was shown to be present across genotypes. Allele frequencies were calculated and tested by test.

### Measurement of serum IGF1 and insulin concentrations

IGF1 and insulin concentrations were measured in serum samples from the cord blood of newborns. IGF1 concentration was measured using a chemiluminescent immunometric assay after pre-treatment with acid using Immulite**®**2000 (Siemens Healthcare Diagnostics Products Llanberis, UK). Insulin concentration was measured using Liaison**®**Insulin (DiaSorin, Saluggia, Italy).

### Statistical analysis

Birth weight (sds) and birth length (sds) were regressed on methylation of CpG-137, sex, folate, supplement intake before conception (yes/no), folate supplement intake during pregnancy (yes/no), parity (primiparous/non primiparous), maternal age, and smoking (yes/no) in multivariate linear regression. Results are expressed as mean ± sd. All statistical analyses were conducted using R 3.3.2.

## Results

### Effects of smoking and folate on birth weight

Six percent of the newborns were from smoking mothers. The mean birth length of neonates born to a smoking mother was 0.62 SDS (95% CI 0.22–1.04) lower than that of neonates born to a non-smoking mother (*P* = 3.0 × 10^−3^), a result retrieved in the multivariate analysis (*P* = 5.6 × 10^−3^). Thirty-two percent of women took a folate supplement before conception. The bivariate analysis showed a trend for increased birth weight associated with folate intake before conception (*P* = 0.09), an association found strengthened in the multivariate regression (*P* = 9.2 × 10^−3^). No association between birth length or birth weight was found with a supplement of folate during pregnancy.

Neither smoking nor folate intake were associated with variations in CpG-137 methylation.

### Epigenetic and genetic variation at the *IGF1* locus

While methylation of CpG-218 located within the *IGF1* P2 promoter correlated closely with methylation of CpG-137 (*P* = 9.4 × 10^−15^), methylation of CpGs -1044, -960, and -919, located within the P1 promoter (Table 2), showed no correlation with CpG-137 methylation (Additional file 3: Figure S2). We found no correlation between the rs35767 genotype and the methylation of CpG-137 of the P2 or other studied CpGs (Additional file 4: Figure S3A).

**Table 2:** CpG methylation (%) in the promoters of the *IGF1* and *INS* genes of the studied newborns

*IGF1* gene Promoter 1	
CpG-1044	89 ± 4%
CpG-960	82 ± 3%
CpG-919	93 ± 9%
*IGF1* gene Promoter 2	
CpG-232	57 ± 8%
CpG-224	67 ± 9%
CpG-218	68 ± 9%
CpG-207	43 ± 6%
CpG-137	49 ± 8%
*INS* gene Promoter	
CpG-180	61 ± 8%
CpG-206	83 ± 5%

**Fig. 1:**
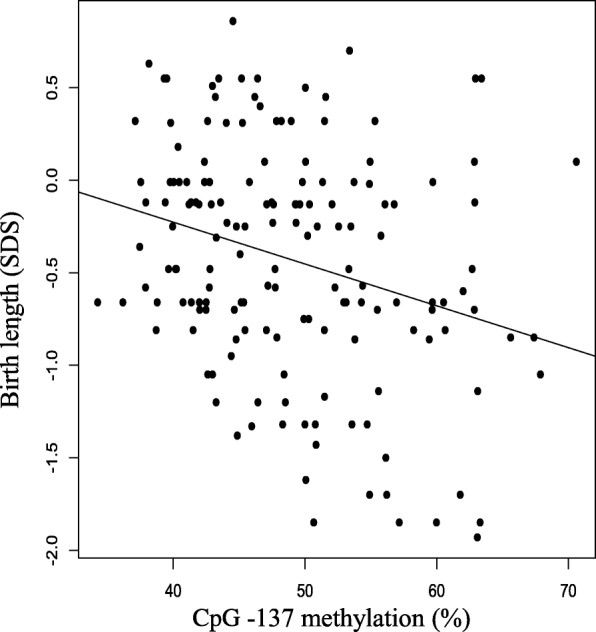
CpG-137 methylation correlates negatively with birth length. The correlation between CpG-137 (%) and birth length (SDS) is described by the equation: Birth length = − 0.023 × [CpG-137 methylation] + 0.68 with *r* = 0.2 and *P* = 3.5 × 10^−4^

Birth length correlated negatively with methylation at CpG-137 (*r* = 0.2, *P* = 3.5 × 10^−4^, Fig. 1). This finding was confirmed after adjustment for several covariates: a 10% increase in methylation at CpG-137 was associated with a decrease of birth length by 0.23 SDS (95% CI 0.11–0.35; *P* = 1.6 × 10^−4^). In contrast, methylation at CpG-137 was not associated with birth weight (*P* = 0.36). The same result was obtained in the multivariate regression framework (*P* = 0.15). Cord blood IGF1 concentration showed no association with methylation at CpG-137 (*r* = 0.06, *P* = 0.61).

Methylation at CpG-224 and CpG-218 correlated negatively with birth length (*P* = 0.03 and *P* = 0.02, respectively) (Additional file 5: Figure S4).

### Epigenetic and genetic variation at the *INS* locus

Methylation of *INS* CpG-180 and CpG-206 showed no correlation with birth length or birth weight. Birth length and birth weight were not associated with rs689 alleles (Additional file 6: Figure S5).

As previously reported [28], we found that CpG-206 and CpG-180 methylation in the studied newborns was strongly influenced by rs689 alleles (Additional file 4: Figure S3B; *P* < 2.10^−16^).

## Discussion

The fetal genotype explains only a limited part of intrauterine growth variability among individuals of the general population [13], which leaves an important role to maternal genotype, maternal environment, and fetal and placental epigenetics. Animal studies have demonstrated that environment can shape the epigenome, notably during the intrauterine period when it has the greatest plasticity. Epigenetic effects of the intrauterine environment can thus influence the phenotype in later life. However, it remains unclear as to how influential the fetal period is in shaping the epigenome, whether different genomic regions show varying sensitivities to this environment during this period, and the extent to which this early interaction is sensitive to genetic influences.

To our knowledge, few studies have examined the association of CpG methylation with fetal growth. The first study found an increased methylation of all CpG sites at the *IGF1* P1 promoter in the placenta of fetuses with IUGR [31], associated with the previously reported decreased *IGF1* gene expression [32]. Another study used the Illumina Infinium HM27 platform to profile CpG methylation in CBMC in a small number of twin pairs [33]. Array technology, however, uses CpGs that are not necessarily those having specific effects on gene expression and phenotypes. This was the case at the *IGF1* locus for the array used in twins, which analyzed only 1 CpG of the P1 promoter and no CpG of the P2 promoter at the *IGF1* locus, while it examined 4 CpGs at the *INS* locus. Findings were that none of these CpGs had their methylation level associated with twins’ birthweight. A third study of methylation restricted to growth-related genes found no association between IUGR and the methylation of CpGs at the *IGF1*, *IGF2*, and *INS* loci in placenta or cord blood samples [24].

Instead, our study focused on CpGs located within the regulatory region of *IGF1* and *INS* genes, because these two genes are known to be major contributors to fetal growth. Some of these CpGs were selected because their methylation was already known to be associated with gene expression [25, 28], the others because they were located within neighboring regulatory regions. We found that CpG-137 methylation was negatively associated with birth length in normal neonates. The methylation of CpG-137 has a strong functional role upon *IGF1* gene expression in children’s PBMC and in the HEK293 cell line [25]. It was previously shown to be associated with postnatal growth variability [13]. If methylation at CpG-137 in CBMC is a proxy of the methylation in growth-promoting tissues of the fetus (which has not been shown in the current study), one can speculate that methylation at this CpG influences *IGF1* gene expression and IGF1 production in fetal tissues, thus fetal growth. There is no evidence, however, that the individual variation of CpG methylation reflects that in growth-promoting tissues. While this is a major weakness of our study, this weakness is shared with a majority of studies on epigenetics in humans where blood cells are used as proxies of physiological tissues, given that clinical research does not have access to these tissues [34]. Another weakness of measuring CpG methylation in CBMC is that it is a unique cell mixture containing red blood cells in addition to other blood cells, and not a well-characterized homogeneous cell type. Our study was not able to estimate cell composition of this mixture based on methods developed for adult blood cells [35], nor to sort a specific cell category from the cord blood sample.

Several hypotheses may account for the association of methylation at CpG-137 measured at birth with fetal growth. A first possibility is that the individual variation in CpG-137 methylation is determined in the post implantatory embryo, at time of the primary shaping of the methylome. Alternatively, the variation observed in the level of methylation of CpG-137 may result from yet unknown maternal signals transmitted to the fetal tissues through the placenta at a post-embryonic stage of intrauterine life. No association of fetal growth was observed with the other CpGs of the *IGF1* locus, except for CpG-218, another CpG of the P2 promoter.

Maternal smoking [36] or folate intake [37] has been shown in other studies to affect methylation of certain CpGs, but was not found to affect the methylation of CpG-137 or of other CpGs studied herein.

Since insulin is a major growth factor in fetal life and the *INS* VNTR has a direct effect on insulin transcription [27], it is conceivable that *INS* VNTR variation influences early growth. This is why CpG-180 and CpG-206 located within the *INS* promoter were selected for the current study, given that their level of methylation affects the expression of the insulin gene [27, 28]. As reported previously [28] the methylation of CpG-206 and CpG-180 was strongly influenced by rs689 alleles. We found that neither rs689 alleles nor CpG-206 or CpG-180 showed a relationship with birth length or birth weight, but this conclusion should await the observation of a large number of neonates. Unlike Dunger et al. [15], we observed no association (Additional file 4: Figure S3) between birth size and insulin VNTR classes or rs689 (in complete linkage disequilibrium with VNTR classes). This may be due to the fact that we were not able to distinguish “non-changers” [15] among the studied neonates: Non-changers is the term used to describe infants that do not show a catch-up growth after being born small for gestational age.

## Conclusions

In conclusion, fetal growth in normal neonates is associated with the methylation of CpG-137 located in the *IGF1* P2 promoter. This observation supports a significant role of IGF1 epigenetics in the regulation of fetal growth that does not seem to be dependent on cis-genetic variation at the IGF1 locus. Although small, this epigenetic effect is of an order of magnitude comparable with that of many genomic variants associated with human quantitative traits.

## Additional files

Additional file 1: **Figure S1.** Schematic representation of the *IGF1* and *INS* loci. (A) The two *IGF1* gene promoters are figured. CpGs are indicated as lollypops (studied CpGs in white and non-studied CpG in black). rs35767 is indicated by a black arrow. TSS are shown as broken arrows. (B) *INS* promoter is figured. CpGs are indicated as lollypops (studied CpGs in white and non-studied CpG in black). TSS is shown as broken arrow. Location of primers are indicated by arrow for CpG methylation and genotyping. Sequences of primers and location on chromosome are provided in Additional file 2: Table S1. (PPTX 67 kb)

Additional file 2: **Table S1.** List of primers and location used in our study. Sequences are given from 5′ to 3′. (DOCX 15 kb)

Additional file 3: **Figure S2.** Correlation matrix of methylation values (%) at the CpG located in the P1 and P2 promoters of the *IGF1* gene in newborns patients. Pearson correlation coefficient is indicated in bold, and *P* value below. (PPTX 88 kb)

Additional file 4: **Figure S3.** Relationship between promoter CpG methylation and genotypes. (A) Methylation at CpGs-137 of the *IGF1* P2 promoter is independent from the rs35767 genotypes. (B) Methylation at CpGs-206 and CpG-180 in insulin promoter is closely dependent on rs689 alleles. (PPTX 242 kb)

Additional file 5: **Figure S4.** Relation between CpG methylation and birth length (SDS) at the *IGF1* promoter 1 and 2. (A) at *IGF1* P1 promoter, we observed no significant correlation of birth length with the studied CpGs, (B) at *IGF1* P2 promoter, only two CpGs other that CpG-137 showed a weak correlation with birth length. The correlation between CpG-224 (%) and birth length (SDS) is described by the equation: Birth length = − 0.014*[CpG-224 methylation] + 0.43 (*r* = 0.17, *P* = 0.03). The correlation between CpG-218 (%) and birth length (SDS) is described by the equation: Birth length = − 0.016*[CpG-218 methylation] + 0.58 (*r* = 0.2, *P* = 0.02). (PPTX 752 kb)

Additional file 6: **Figure S5.** Relationship between *insulin* rs689 genotype and birth weight (sds) and birth length (sds). Birth weight (sds) and birth length (sds) are independent from the rs 689 genotypes. (PPTX 161 kb)

## Abbreviations

CBMC: Cord blood mononuclear cells
IGF1: Insulin-like growth factor 1
IUGR: Intrauterine growth retardation
