# Correction to: Fetal growth is associated with CpG methylation in the P2 promoter of the *IGF1* gene

**DOI:** 10.1186/s13148-018-0506-z

**Published:** 2018-06-04

**Authors:** Catherine Le Stunff, Anne-Laure Castell, Nicolas Todd, Clémence Mille, Marie-Pierre Belot, Nathalie Frament, Sylvie Brailly-Tabard, Alexandra Benachi, Delphine Fradin, Pierre Bougnères

**Affiliations:** 1 Institut National de la Santé et de la Recherche Médicale U1169, Bicêtre Hospital, Paris Sud University, Le Kremlin-Bicêtre, France; 2 Service de Médecine des Adolescents, Bicêtre Hospital, Paris Sud University, Le Kremlin-Bicêtre, France; 3 Service de BiologieMoléculaire et Hormonologie, Bicêtre Hospital, Paris Sud University, Le Kremlin-Bicêtre, France; 4 Service de Gynécologie-Obstétrique, Antoine Béclère Hospital, Paris Sud University, Clamart, France; 5 CRCINA, INSERM U1232, Université de Nantes, Nantes, France

## Correction

After publication of the original article [1], it came to the publishers’ attention that the below author’s corrections provided at the proofing stage had been misinterpreted.

1. The title should have read “Fetal growth is associated with CpG methylation in the P2 promoter of the *IGF1* gene”, and not “Fetal growth is associated with the CpG methylation of the P2 promoter of the IGF1 gene”.
2. The first sentence of Fig. 1 caption should have read “CpG-137 methylation correlates negatively with **birth length**”, and not “CpG-137 methylation correlates negatively with **height**”.
3. Additional file 3 legend should have read “Correlation matrix of methylation values (%) at the **CpG** located in the P1 and P2 promoters of the IGF1 gene in newborns patients. Pearson correlation coefficient is indicated in bold, and P value below.”, and not “Correlation matrix of methylation values (%) at the **CG** located in the P1 and P2 promoters of the IGF1 gene in newborns patients. Pearson correlation coefficient is indicated in bold, and P value below.”
4. Additional file 4 legend should have read “Relationship between promoter **CpG** methylation and genotypes. (A) Methylation at CpGs-137 of the IGF1 P2 promoter is independent from the rs35767 genotypes. (B) Methylation at CpGs-206 and CpG-180 in insulin promoter is closely dependent on rs689 alleles.”, and not “Relationship between promoter **CG** methylation and genotypes. (A) Methylation at CpGs-137 of the IGF1 P2 promoter is independent from the rs35767 genotypes. (B) Methylation at CpGs-206 and CpG-180 in insulin promoter is closely dependent on rs689 alleles.”

The original article has been updated to reflect these corrections.
