# Supplementary material for: Fetal growth is associated with CpG methylation in the P2 promoter of the IGF1 gene
Source: Clin Epigenetics. 2018 Apr 19;10:57. doi: 10.1186/s13148-018-0489-9 (PMC5909239; doi:10.1186/s13148-018-0489-9)
Supplement: Supplementary file 1 — Figure S1. Schematic representation of the IGF1 and INS loci. (A) The two IGF1 gene promoters are figured. CpGs are indicated as lollypops (studied CpGs in white and non-studied CpG in black). rs35767 is indicated by a black arrow. TSS are shown as broken arrows. (B) INS promoter is figured. CpGs are indicated as lollypops (studied CpGs in white and non-studied CpG in black). TSS is shown as broken arrow. Location of primers are indicated by arrow for CpG methylation and genotyping. Sequences of primers and location on chromosome are provided in Additional file 2: Table S1. (PPTX 67 kb) [file 13148_2018_489_MOESM1_ESM.pptx]

## Slide 1
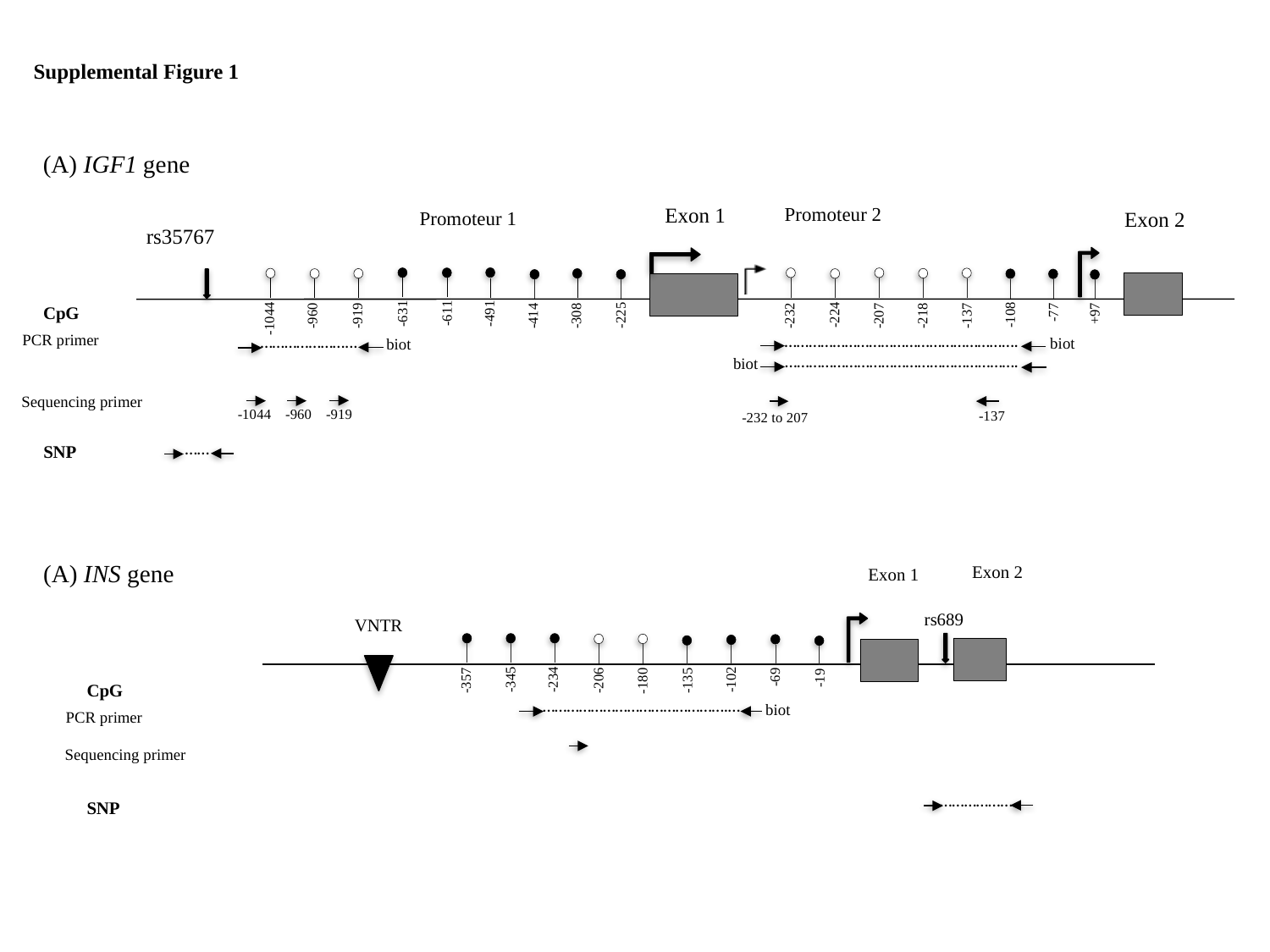

Supplemental Figure 1
(A) IGF1 gene
Exon 1
Promoteur 2
Promoteur 1
Exon 2
rs35767
-225
-414
-611
-491
-960
-919
-308
-77
-108
-224
-137
-1044
-207
-232
+97
-218
-631
CpG
………………………………………………….
PCR primer
…………………...
biot
biot
………………………………………………….
 biot
Sequencing primer
-1044 -960 -919
-137
-232 to 207
……
SNP
(A) INS gene
Exon 2
Exon 1
rs689
VNTR
-69
-19
-102
-345
-234
-206
-357
-135
-180
CpG
……………………………………….…
biot
PCR primer
Sequencing primer
………………
SNP
