# Supplementary material for: Fetal growth is associated with CpG methylation in the P2 promoter of the IGF1 gene
Source: Clin Epigenetics. 2018 Apr 19;10:57. doi: 10.1186/s13148-018-0489-9 (PMC5909239; doi:10.1186/s13148-018-0489-9)
Supplement: Supplementary file 2 — Table S1. List of primers and location used in our study. Sequences are given from 5′ to 3′. (DOCX 15 kb) [file 13148_2018_489_MOESM2_ESM.docx]

| **Table S1**: List of primers and location used in our study. Sequences are given from 5’ to 3’. | |
| --- | --- |
| **IGF1 gene** |  |
| *Pyrosequencing Promoter 1* |  |
| CpGS -1044 to -919 | F- TATTGTTTTATAAAATTAAAGGGAA |
|  | R- ATTCTAAATAACACCAACTAACTAAC |
| Sequencing CpG -1044 | F- AGGGAAATAGGTATAAAT |
| Sequencing CpG -960 | F- AGTTGGTTTGGATTATGT |
| Sequencing CpG -919 | F- GGGATTTGAATGATATTA |
| *Pyrosequencing Promoter 2* |  |
| CpGs -232 to 108 | F- AATTTGGTTGTTGTTGTTAGTGTAT |
|  | R- AATTAAACCCTCAAACAATTAAATC |
| Sequencing CpGs -232, -224, -218 and -207 | F- TGGTTGTTGTTGTTAGTGTAT |
| Sequencing CpG -137 | R - ACCAATAACAACAACTTAA |
| *Genotyping* |  |
| rs35767 | F - GGATTTCAAGCAGAACTGTGTTTTCA R - GGTGGAAATAACCTGGACCTTGAAT F - VIC-TTTTTTCCGCATGACTCT R - FAM-TTTTTTTTCCACATGACTCT |
| *Chromosomal Location (hg19)* |  |
| PCR for CpGs-1044 to -919 | chr12:102875221-102875428 |
| PCR for CpGs -232 to 108 | chr12:102873120-102873294 |
| rs35767 | chr12:102875569 |
| PCR for rs35767 | chr12 :102523-102875624 |
| **Insulin gene** |  |
| *Pyrosequencing* |  |
| CpGs -206 and -180 | F- TGTGAGTAGGGATAGGTTTGGTTAT |
|  | R- CCATTAAAACCTAAAATAAAAAAATC |
| Sequencing CpGs -206 and -180 | F- TTTTGGTTAAGATTTTAATG |
| *Genotyping* |  |
| rs689 | F- TCCAGGACAGGCTGCATCAG |
|  | R- AGCAATGGGCGGTTGGCTCA |
| *Chromosomal Location (hg19)* |  |
| rs689 | chr11:2182224 |
| Region for studied CpGs | chr11:2182515-2182698 |
| PCR for rs689 | chr11:2181992-2182435 |
