# Supplementary figures and images for: Fetal growth is associated with CpG methylation in the P2 promoter of the IGF1 gene
Source: Clin Epigenetics. 2018 Apr 19;10:57. doi: 10.1186/s13148-018-0489-9 (PMC5909239; doi:10.1186/s13148-018-0489-9)

## Slide 1
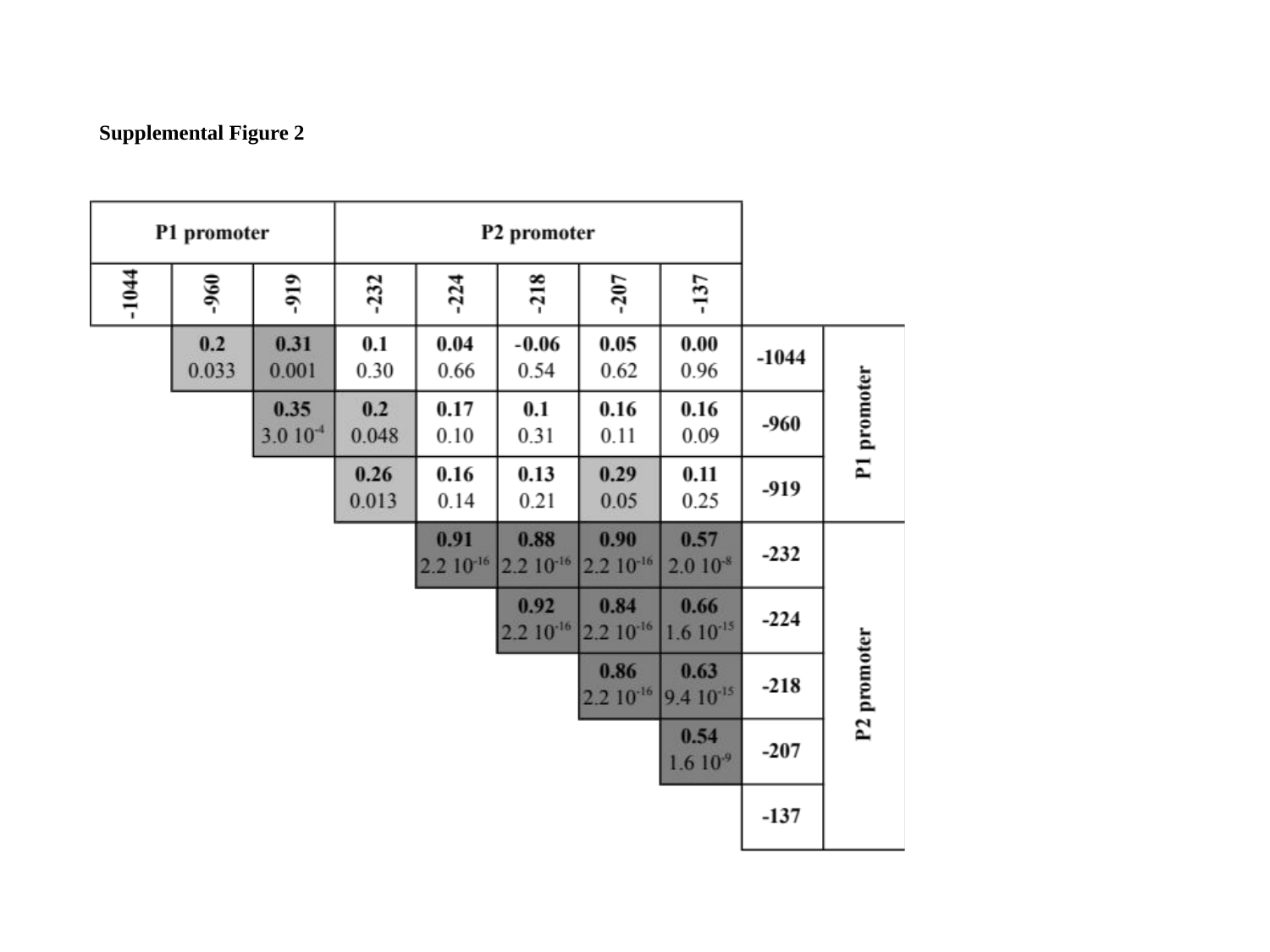

Supplemental Figure 2

Supplement: Supplementary file 3 — Figure S2. Correlation matrix of methylation values (%) at the CpG located in the P1 and P2 promoters of the IGF1 gene in newborns patients. Pearson correlation coefficient is indicated in bold, and P value below. (PPTX 88 kb) [file 13148_2018_489_MOESM3_ESM.pptx]
