# Supplementary material for: Fetal growth is associated with CpG methylation in the P2 promoter of the IGF1 gene
Source: Clin Epigenetics. 2018 Apr 19;10:57. doi: 10.1186/s13148-018-0489-9 (PMC5909239; doi:10.1186/s13148-018-0489-9)
Supplement: Supplementary file 4 — Figure S3. Relationship between promoter CpG methylation and genotypes. (A) Methylation at CpGs-137 of the IGF1 P2 promoter is independent from the rs35767 genotypes. (B) Methylation at CpGs-206 and CpG-180 in insulin promoter is closely dependent on rs689 alleles. (PPTX 242 kb) [file 13148_2018_489_MOESM4_ESM.pptx]

## Slide 1
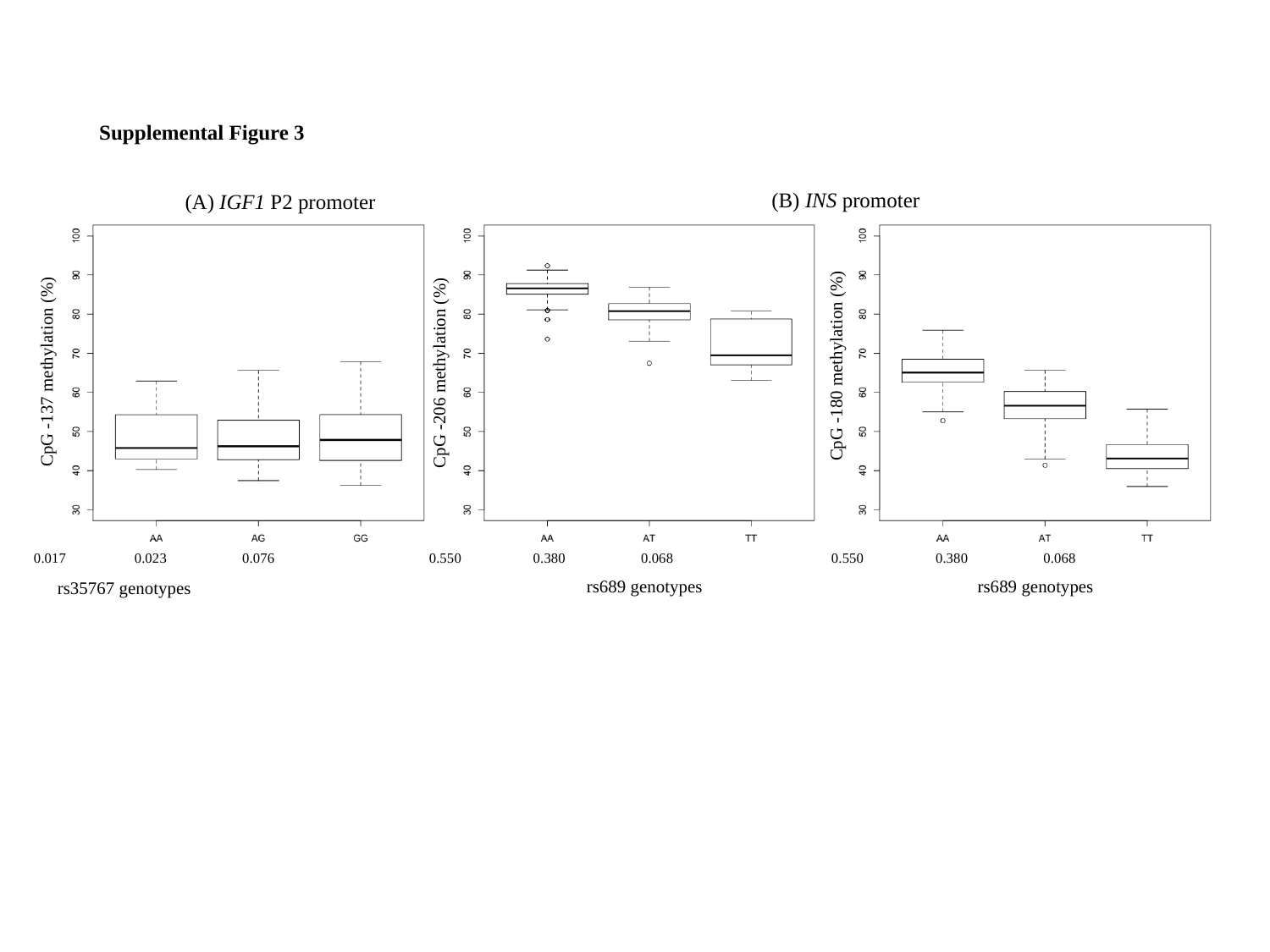

Supplemental Figure 3
(B) INS promoter
(A) IGF1 P2 promoter
CpG -180 methylation (%)
CpG -137 methylation (%)
CpG -206 methylation (%)
Allele 0.017 0.023 0.076 0.550 0.380 0.068 0.550 0.380 0.068
Frequency
rs689 genotypes rs689 genotypes
rs35767 genotypes
