# Supplementary material for: Fetal growth is associated with CpG methylation in the P2 promoter of the IGF1 gene
Source: Clin Epigenetics. 2018 Apr 19;10:57. doi: 10.1186/s13148-018-0489-9 (PMC5909239; doi:10.1186/s13148-018-0489-9)
Supplement: Supplementary file 5 — Figure S4. Relation between CpG methylation and birth length (SDS) at the IGF1 promoter 1 and 2. (A) at IGF1 P1 promoter, we observed no significant correlation of birth length with the studied CpGs, (B) at IGF1 P2 promoter, only two CpGs other that CpG-137 showed a weak correlation with birth length. The correlation between CpG-224 (%) and birth length (SDS) is described by the equation: Birth length = − 0.014*[CpG-224 methylation] + 0.43 (r = 0.17, P = 0.03). The correlation between CpG-218 (%) and birth length (SDS) is described by the equation: Birth length = − 0.016*[CpG-218 methylation] + 0.58 (r = 0.2, P = 0.02). (PPTX 752 kb) [file 13148_2018_489_MOESM5_ESM.pptx]

## Slide 1
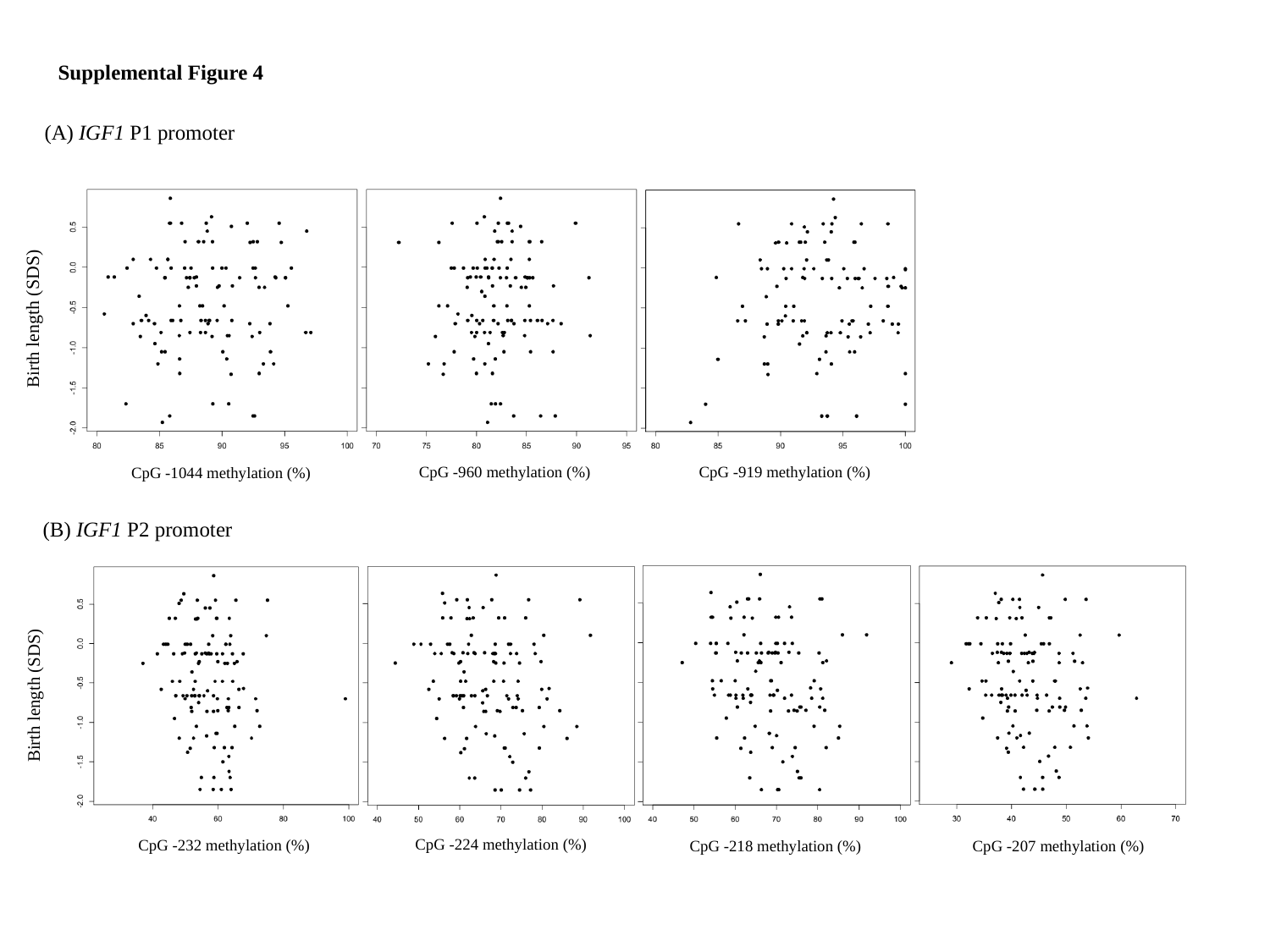

Supplemental Figure 4
(A) IGF1 P1 promoter
Birth length (SDS)
CpG -960 methylation (%)
CpG -919 methylation (%)
CpG -1044 methylation (%)
(B) IGF1 P2 promoter
Birth length (SDS)
CpG -224 methylation (%)
CpG -232 methylation (%)
CpG -207 methylation (%)
CpG -218 methylation (%)
