# Supplementary material for: Fetal growth is associated with CpG methylation in the P2 promoter of the IGF1 gene
Source: Clin Epigenetics. 2018 Apr 19;10:57. doi: 10.1186/s13148-018-0489-9 (PMC5909239; doi:10.1186/s13148-018-0489-9)
Supplement: Supplementary file 6 — Figure S5. Relationship between insulin rs689 genotype and birth weight (sds) and birth length (sds). Birth weight (sds) and birth length (sds) are independent from the rs 689 genotypes. (PPTX 161 kb) [file 13148_2018_489_MOESM6_ESM.pptx]

## Slide 1
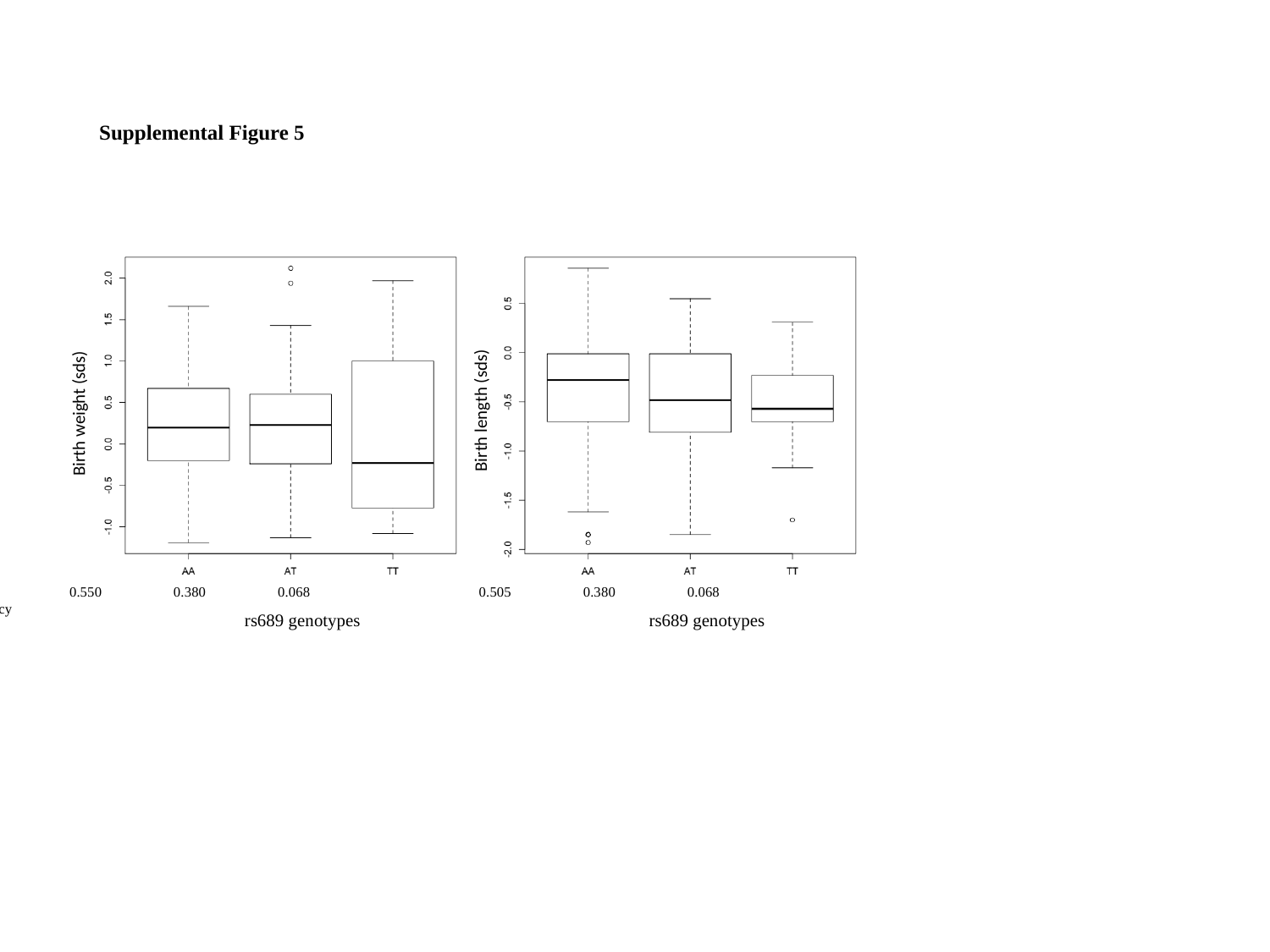

Supplemental Figure 5
Birth length (sds)
Birth weight (sds)
rs689 genotypes rs689 genotypes
 Allele 0.550 0.380 0.068 0.505 0.380 0.068
 Frequency
